# Vaping Possible Negative Effects on Lungs: State-of-the-Art From Lung Capacity Alteration to Cancer

**DOI:** 10.7759/cureus.72109

**Published:** 2024-10-22

**Authors:** Fakher Rahim, Karlygash Toguzbaeva, Dmitriy Sokolov, Kenesh O Dzhusupov, Abzal Zhumagaliuly, Ainur Tekmanova, Elmira Kussaiynova, Aiya Katayeva, Sholpan Orazbaeva, Aidana Bayanova, Mariyam Olzhas, Alina Zhumataeva, Sabina Moldabekova

**Affiliations:** 1 Department of Medical Laboratory Technologies, Alnoor University, Mosul, IRQ; 2 Department of Public Health, Asfendiyarov Kazakh National Medical University, Almaty, KAZ; 3 Public Health Sciences, International Higher School of Medicine, Bishkek, KGZ; 4 Biology, Haileybury Astana School, Astana, KAZ; 5 Biology, School-Gymnasium #22, Astana, KAZ

**Keywords:** cancer, electronic cigarettes, lung function, lung injury, vape

## Abstract

Vaping has emerged as a popular alternative to traditional smoking. It produces smokeless vapour by heating an e-liquid mixture in an atomizer. This paper delves into the current state of knowledge surrounding electronic cigarettes, exploring the gap between the perceived safety of e-liquids and the emerging evidence of their harmful effects when inhaled. As we navigate this intricate landscape, it is crucial to unravel the complexities of vaping and its implications for public health.

We conducted a three-layer systematic review of the guidelines set by the Preferred Reporting Items for Systematic Reviews and Meta-analyses (PRISMA) and Meta-analyses of Observational Studies in Epidemiology (MOOSE). The search was performed in three layers, including the first layer, the effect of vaping on lung function; the second layer, the effect of vaping on lung structure and inducing lung injury; and the third layer, the physiopathologic effect of vaping on the lung and a possible carcinogenic effect.

Exposure to e-cigarette vapour reduced lung ventilation in adult male Long-Evans rats, indicating impaired lung function. In male Wistar rats, vaping was associated with a decrease in lung air volume and denser lung tissue structure. Studies on guinea pigs showed that vaping caused acute bronchoconstriction, contributing to lung function impairment.

A case study of a young man with an E-cigarette and vaping-induced lung injury (EVALI) highlighted the detrimental effects of vaping on human lung function. The EVALI outbreak in the USA was linked to harmful substances in vapes, such as vitamin E acetate and THC, leading to serious lung injuries, including pneumonia and bronchiolitis. Vaping poses significant health risks, especially to young adults, and misconceptions regarding its safety persist despite evidence of its potential to cause various lung diseases.

While vaping has positioned itself as a smoking cessation aid, the discussion surrounding its impact on lung health requires careful consideration. The lack of conclusive evidence on the long-term effects of vaping underscores the need for further research. However, existing data suggest that vaping is not without risks, and its potential association with respiratory issues and cancer underscores the urgency of public health interventions.

## Introduction and background

Electronic cigarettes, commonly referred to as vape pens, have emerged as a popular alternative to traditional smoking, producing smokeless vapour by heating an e-liquid mixture in an atomizer [1]. The e-liquid, while deemed safe for oral ingestion [2], raises concerns when inhaled as an aerosol. Since its inception in 2003, vaping has consistently increased in popularity [3]. Despite claims suggesting that vaping is less harmful than smoking, a growing body of evidence establishes links between vaping and various adverse health outcomes [4].

Contrary to the perceived safety of e-cigarettes, the Centres for Disease Control and Prevention (CDC) reported 2,807 cases of pulmonary damage associated with e-cigarette or vaping product use during hospital stays in early 2020 [5]. A 2019 study revealed alarming symptoms in individuals hospitalized due to e-cigarette or vaping-related lung damage, including decreased blood oxygen levels, elevated body temperature, inflammatory responses, and abnormal lung opacities observed on imaging scans [6].

Studies have shown a significant increase in vaping among youth, with a National Youth Tobacco Survey finding that 4.9% of middle school students and 20.8% of high school students used e-cigarettes within the past 30 days as of 2018 [7]. This demographic is particularly at risk of E-cigarette and vaping-induced lung injury (EVALI), highlighting the urgent need for public health interventions. This accumulating evidence challenges the notion that vaping is a risk-free substitute for traditional cigarette smoking.

In this context, it is imperative to critically examine the existing literature on the chemistry and toxicology of vaping and shed light on the potential health risks associated with this rapidly growing phenomenon [1]. This paper delves into the current state of knowledge surrounding e-cigarettes, exploring the gap between the perceived safety of e-liquids and the emerging evidence of their harmful effects when inhaled. As we navigate this intricate landscape, it is crucial to unravel the complexities of vaping and its implications for public health.

## Review

Methods

Design

We conducted a three-layer systematic review of the guidelines set by the Preferred Reporting Items for Systematic Reviews and Meta-analyses (PRISMA) [8]. This review was not pre-registered, and the study results should be considered exploratory.

Search Strategy

**Figure 1:**
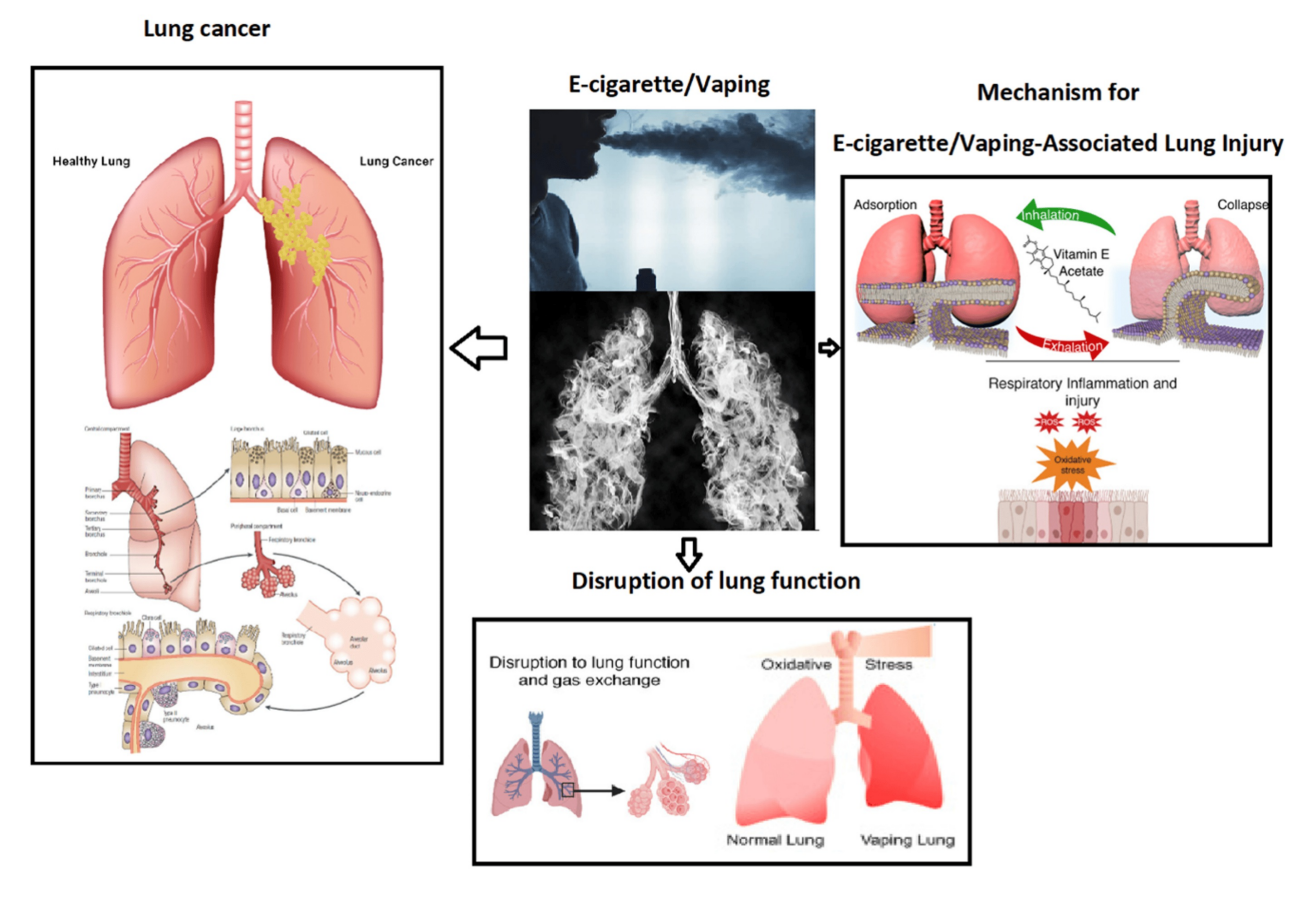
A schematic illustration of the possible effects of vaping or e-cig on the lungs and respiratory system Original illustration, made by the author, F. Rahim

The search on PubMed was performed in three layers: the first layer, the effect of vaping on lung function; the second layer, the effect of vaping on lung structure and inducing lung injury; and the third layer, the physiopathologic effect of vaping on lung and possible carcinogenic effect (Figure 1).

First layer: This layer is concerned with the association between vaping and lung function. We used keywords including ((("ERV"[Title/Abstract] OR "expiratory reserve volume" [Title/Abstract] OR "FEFmax"[Title/Abstract]) AND ("FRC"[Title/Abstract] OR "functional residual capacity"[Title/Abstract])) OR ("FVC"[Title/Abstract] OR "forced vital capacity"[Title/Abstract])) AND ("IC"[Title/Abstract] OR "inspiratory capacity"[Title/Abstract]) to find all available evidence on the effect of vape or electronic cigarette on lung function and capacities.

Second layer: This layer was about the association between vaping and lung injury. The search was performed using specific keywords (((((vape [Title/Abstract]) OR (electronic cigarette [Title/Abstract])) OR (vaping [Title/Abstract])) OR (e-cigarette [Title/Abstract])) AND ((lung [Title/Abstract]) AND (injury [Title/Abstract]))), covering the period from July 15, 2015, to December 2023, without any language limitations. EVALI cases were first reported to the Centres for Disease Control and Prevention (CDC) in August 2019 and rapidly increased thereafter, suggesting new or increased exposure to one or more toxicants from the use of e-cigarette products [9].

Third layer: This layer was about the association between vaping and lung cancer. We also used keywords such as ("vape"[Title/Abstract] OR "electronic cigarette"[Title/Abstract] OR "vaping"[Title/Abstract] OR "e-cigarette"[Title/Abstract]) AND ("cancer"[Title/Abstract] OR "neoplasm"[Title/Abstract] OR "tumorigenic"[Title/Abstract]) to find all available evidence on the effect of vape or electronic cigarette on pathology or physiology and ultimately possible inducing cancer.

The inclusion criteria were as follows: (i) Study types included are original research articles, including observational studies (cohort, case-control, and cross-sectional), randomized controlled trials, and case reports that investigate the effects of vaping on lung health; (ii) subjects: studies involving humans of any age, sex, and health status; (iii) measured parameter: articles that assess lung function, structural lung injury, physiopathological effects, and potential carcinogenic outcomes related to vaping; (iv) publication language: studies published in English; (v) date of publication: studies published between July 15, 2015, and December 2023.

The exclusion criteria were as follows: (i) study types: reviews, editorials, commentaries, and letters to the editor; (ii) subjects: studies on animals, unless the findings are directly related to human health implications and are used to supplement human data; (iii) measured parameters: articles that do not directly assess the impact of vaping on lung health, such as those focusing solely on the chemical composition of e-liquids without linking to health outcomes; (4) duplicate studies and studies with incomplete data or those lacking clear methodology and results.

Results

**Figure 2:**
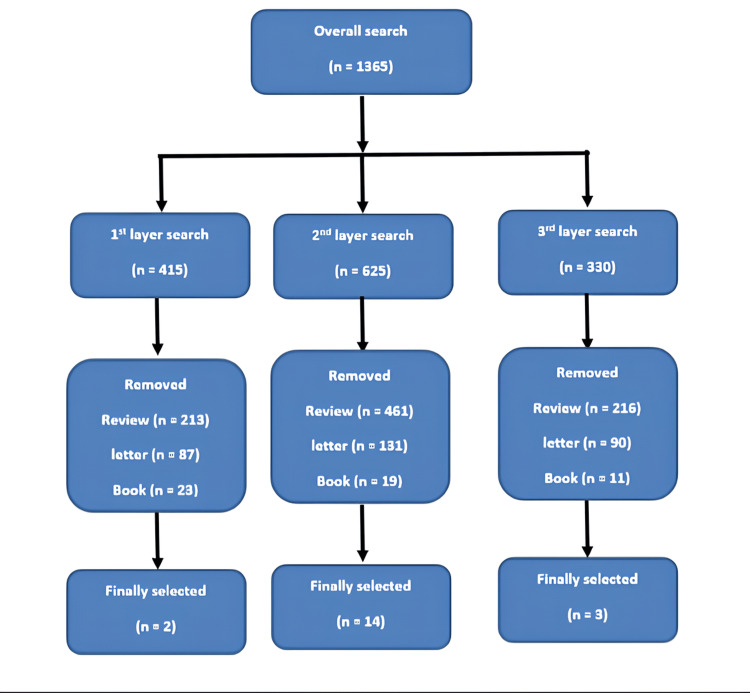
Flow diagram of search strategy

We found 1365 articles in a three-layer search, including 415 on the association between vaping and lung function, 625 on the association between vaping and lung injury, and 330 on the association between vaping and lung cancer (Figure 2). Finally, after removing duplicates, reviews, and letters, 19 articles were selected, including two on the association between vaping and lung function, 14 on the association between vaping and lung injury, and 3 on the association between vaping and lung cancer.

Vape and Lung Capacity

Four studies investigated the association between vaping and lung function overall (Table 1). In a study by Stokes and Fisher, adult male Long-Evans rats exposed to e-cigarette vapour for ten minutes exhibited a reduction in both inhaled and exhaled air per minute, indicating a decrease in overall lung ventilation [10]. This reduction in tidal volume might be attributed to protective bronchoconstriction, as observed by Khosravi et al. in adult guinea pigs exposed to vapour [11]. Furthermore, Odish et al. reported the case of a 19-year-old man with EVALI, where the history and imaging findings were consistent with lung injury caused by e-cigarette use, emphasizing the negative effects of vapour on lung function [12]. Lee et al. conducted a study on young, male Hartley guinea pigs [13]. They revealed that vaping induced acute bronchoconstriction, contributing to the impairment of lung function. Yanina et al. observed changes in the morphological and optical properties of lung tissue in male Wistar rats, further supporting the notion that vaping leads to reduced lung function [14].

**Table 1:** Comparing findings of studies on the effects of vaping on lung capacity

Study ID	Study type	Target group	Findings	Interpretation
Stokes and Fisher [10]	Animal model	18 adult male Long-Evans rats	Tidal volume ↓ Minute ventilation↓	Vaping leads to reduced lung function
Yanina et al. [14]	Animal model	12 sexually mature male Wistar rats	the volume of air in the lung ↓ packing of lung structures ↑	Vaping leads to reduced lung function
Odish et al. [12]	Human	A 19-year-old man	Tidal volume ↓	Vaping leads to reduced lung function
Lee et al. [13]	Animal model	Young, male Hartley guinea pigs	dynamic lung compliance (C_dyn_) ↓	Vaping affects lung function by evoking acute bronchoconstriction

Vape and Lung Injury

Our search returned 14 studies that discussed the association between vaping and lung injury (Table 2). Research indicates that e-cigarettes, especially those containing nicotine, disturb mitochondrial membrane potential, release ATP and mitochondrial DNA (mtDNA), and trigger inflammatory responses [15]. Vitamin E acetate (VEA) is implicated in EVALI. It has been discovered to be a diluting agent in illegal vapour pens. In contrast, nicotine is typically diluted with equal amounts of propylene glycol (PG) and vegetable glycerine (VG) [16]. Vaping has become the preferred method of nicotine consumption among young adults (15-24 years old), surpassing traditional cigarettes [17]. Studies conducted in a laboratory setting have shown that the survival of normal human bronchial epithelial cells decreases in a manner that is dependent on the dosage when exposed to vapour emitted by electronic vapour devices [18]. In 2018, the National Youth Tobacco Survey found that 4.9% of students in middle school and 20.8% of students in high school had used e-cigarettes within the past 30 days [18].

**Table 2:** Comparing findings of studies on the association between vaping and lung injury Human pulmonary alveolar epithelial cells (haemic); electronic cigarette (e-Cig); estrogen (ER); E-cigarette and vaping-induced lung injury (EVALI); acute lung injury (ALI); Invasive pulmonary aspergillosis (IPA); ground-glass opacity (GGO); tetrahydrocannabinol (THC)

Study ID	Study design	Study type	Number of cases	Target group	Symptoms	Findings	Diagnosis	Interpretation
Itoh et al. [19]	Case-report	Human	1	A 46-year-old man	Night sweats, fever, weight loss, pallor	Bilateral GGO	ALI	Vaping leads to EVALI
Layden et al. [20]	Case-report	Human	98	15-53-years-old	Shortness of breath, cough, chest pain, nausea, subjective fever	Opacities in both lungs	Lung injury	Vaping leads EVALI
Adhikari et al. [21]	Case-report	Human	1	A 23-year-old	Fever, shortness of breath, tachypnea, nausea, diarrhea	Bilateral infiltrates	Sepsis, pneumonia, lung injury	Vaping leads to EVALI
Sharma et al. [22]	Case-report	Human	1	A 35-year-old	Right-sided chest pain and dyspnea	Ground glass opacity in both lungs	Vaping-induced pneumonitis	pneumothorax due to vape/E-cigarette induced EVALI
Szafran et al. [23]	RCT	Animal	33-36	6-week-old female mice	-------------	Markers associated with lung immunotoxicity ↑ Alterations in lung cell immunophenotyping, and immunosuppression; lung tissue resistance	--------------	Exposures to only e-cig without nicotine, affect the lungs.
Wolf and Richards [24]	Case-report	Human	1	A 29-year-old female	Profound fatigue, fevers, shortness of breath, respiratory distress	Diffuse, broncho-centric bilateral ground glass opacities with coalescence to consolidation, largely in the lung bases	Acute eosinophilic pneumonia	The use of *e*-*Cig* for vaping THC affects acute eosinophilic pneumonia
Kupelian et al. [25]	Case-report	Human	1	A 16-year-old man	Temperature was 37.8 °C, respiratory rate 44 breaths per minute	Severe respiratory distress, and auscultation of the lungs revealed bilateral decreased breath sounds, bilateral hazy ground-glass opacities	IPA as a complication of EVALI	THC and other potential contaminants in vaping affect the lungs in the form of EVALI
Stein et al. [26]	Case-report	Human	1	A 18-year-old man	Shortness of breath, cough or chest pain	Subjective fever, leukocytosis and bilateral opacities of the "frosted glass" type on CT	EVALI	Vaping with THC leads to EVALI
Heinzerling et al. [27]	Case report, interview	Human	160	14-70-years-old	Cough, shortness of breath, and subjective fever or chills	--------------	EVALI	EVALI indicates *e*-*Cig*, or vaping, produce lung injury
Smith et al. [28]	Case-report	Human	1	A 14-year-old girl	Cough, chest discomfort, abdominal pain, and rigors	Diffuse interstitial markings with hazy patchy nodular infiltrates bilaterally	Acute lung injury with centrilobular nodules consistent	The harmful effects of vaping, especially in young people
Yingchoncharoen et al. [29]	Case-report	Human	1	An 18-year-old woman in her 10th week of pregnancy	Productive cough, and dyspnea and a one-day history of left-sided chest pain	Reduction of air intake into the upper and middle zone of the left lung. left-sided pneumothorax	EVALI	Vaping induced lung injuries in association with pregnancy and multiple subsequent viral and bacterial infections
Berkelhamer et al. [30]	Clinical trial	Animal	2	Newborn and adult sheep	------------------	Relaxation of bronchial rings, contraction of the smooth muscles of the respiratory tract	-----------	Gestational and postnatal exposure to electronic cigarettes represents rapidly growing threat
Thakrar et al. [31]	Case report	Human	12	Ten male and two female	Dyspnea, abdominal pain and constitutional symptoms	Centrilobular ground-glass nodules, confluent ground-glass opacities, pleural effusions	ALI	EVALI
Messina et al. [32]	Case report	Human	6	15-20-years-old	Fever, emesis, nausea, abdominal pain, chest pain, headache	Confluent pulmonary opacities, mediastinal/hilar lymphadenopathy and/or small bilateral pleural effusions (3)	EVALI	Vaping leads to EVALI

We reviewed various data regarding the possible effects of vaping/e-cigarettes on lung injury. And as it turned out, in 2019 in the USA, there was an epidemic of EVALI (e-cigarette, or vaping, product use-associated lung injury). Most researchers and doctors agree on the toxic composition of these devices. Vitamin E acetate and/or tetrahydrocannabinol can affect the appearance of pneumonia, broncho-centric bilateral ground glass opacities, and general deterioration of lung function. It is important to note that a clear link has been established between the use of tetrahydrocannabinol in e-cigarettes and EVALI. In addition, as a result of the disease, some patients developed complications in the form of other diseases, such as invasive pulmonary aspergillosis and acute eosinophilic pneumonia. The CDC emphasizes bronchoalveolar lavage (BAL) fluid analysis to detect harmful substances at the presumed site of lung injury [9]. Vitamin E acetate in vapes/e-cigarettes presumably enters the respiratory epithelial-lining fluid, which is a suspected site for lung injury. Its effect is established by the method of detecting vitamin E acetate in BAL. Blount et al. found that 48 out of 51 patients with lung injuries had vitamin E acetate present in the BAL fluid [9]. It is important to note that 99 healthy participants, according to Blount et al., did not find vitamin E acetate in the BAL liquid [9]. Itoh et al. diagnosed e-cig-induced acute lung injury (ALI) caused by using e-cigarettes [19]. A 46-year-old man was diagnosed with ALI due to e-cig use [19]. Lung histological examination revealed lesions with acute changes, alveolar septum swelling, and eosinophil and neutrophil invasion, with intra-alveolar invasion of eosinophils and neutrophils; in addition, abundant macrophages containing blackish-brown pigment, multinucleated foreign-body giant cells, and intra-alveolar organization [19]. It is considered that these changes are due to the ingestion of foreign substances in the composition of the e-cigarette into the respiratory system. Layden et al. presented the results: of the 91 patients who underwent CT imaging, 6 cases of pneumomediastinum, 11 cases of pleural effusion, and 2 cases of pneumothorax were present (in 15 patients) [20]. Two patients had both a pneumomediastinum and a pneumothorax, and two patients had both a pneumomediastinum and pleural effusion [20]. Adhikari et al., in the case report, presented the results of a 23-year-old man [21]. A chest X-ray showed bilateral pneumonia, and a computed tomography (CT) scan of the chest showed bilateral lung infiltrates [4]. In another case report described by Sharma et al., a chest X-ray of a 35-year-old man was examined, which showed a right-sided pneumothorax with a slight displacement of the structures of the heart and mediastinum to the left [22]. Szafran et al. did a study on mice, and it showed that exposure to only e-cig delivery vehicles, VG/PG, without nicotine, affects the lungs and that the addition of vanilla flavouring may enhance the lung responses [23]. There was also a case in the Wolf and Richards et al. report where attention was drawn to acute eosinophilic pneumonia as a potential consequence of lung injury associated with vaping with THC [24]. It is worth mentioning another case report where a teenager was diagnosed with invasive pulmonary aspergillosis as a complication after EVALI. Kupelian et al. reported the first case, most likely related to the use of tetrahydrocannabinol and/or vaping [25]. In the case report of Stein et al., an interesting case was written about a man with testicular cancer who, during the course of the disease, had bilateral opacities of the "frosted glass" type and intrathoracic adenopathy revealed on a CT scan [26]. Later, it turned out that the patient smoked THC three times a day. As mentioned, the epidemic of EVALI is associated with the use of tetrahydrocannabinol and vitamin E acetate (VEA) in e-cigarettes. Heinzerling et al. point out that most foods that contain THC also contain VEA, which reinforces the link between these chemicals and the outbreak [27]. Vaping with tetrahydrocannabinol significantly increases the risk of lung injury and complications after e-cigarette or vaping product use-associated lung injury (EVALI). Besides the above-mentioned EVALI outcomes, other complications may occur, such as pneumothorax and acute respiratory distress syndrome (ARDS). They are mentioned in the case reports by Smith et al. [28] and Yingchoncharoen et al. [29]. ARDS is a life-threatening lung injury characterized by the rapid onset of widespread inflammation in the lungs. Multiple risk factors, including pneumonia, non-pulmonary sepsis, aspiration of gastric contents, or inhalation injury, have been reported to cause ARDS [29]. Currently, unfortunately, some women use e-cigarettes during pregnancy with the opinion that they are harmless. However, due to the similar development and physiology of sheep and human lungs, Berkelhamer et al. conducted a test on adult sheep and their two-day-old lambs in their study [30]. Their data show that foetal and newborn lungs may have increased susceptibility to toxicity when exposed to flavoured e-cigarette solutions and that physiological responses to these common additives may also be altered in immature lungs. We concluded that the use of electronic cigarettes in the foetal period carries a high risk to the foetus. Also, many articles note the bilateral infiltrates, bilateral opacities, and/or ground glass opacity (GGO) in the lungs as a result of smoking e-cigarettes and/or vaping [31,32]. The frightening thing is that in addition to this, the disease is accompanied by various respiratory symptoms and other functional abnormalities with frequent sub-pleural sparing, and small pleural effusions may also be detected. After studying all these articles to identify the link between vaping and lung injury, we conclude that e-cigarette smokers and/or vapers are susceptible to various lung diseases. In particular, due to the legalization of THC in some US states, the situation has accepted and is accepting severe outcomes of smoking electronic cigarettes as well as vaping, which contains vitamin E acetate. Currently, the vaping situation is deteriorating, and it may be a new pandemic, especially among young people and adolescents. Despite the law on the sale of smoking systems only after adulthood, vape shops are not limited to them. Many people mistakenly think that e-cigarettes and vaping are harmless compared to tobacco. However, as can be seen from our review article, the impact is significant and may increase.

Vaping and Lung Cancer

Only two studies were about the association between vaping and lung cancer (Table 3 [PNLP1] and [PNLP2]). Lung cancer (LC) is a diverse disease with different clinical and pathological features. It can be histologically classified into two groups: non-small-cell lung cancer (NSCLC) and small-cell lung cancer (SCLC) [33]. The diagnostic rate for NSCLC is 85%, while for SCLC it is only 15% [34]. Concerning the hereditary predisposition to lung cancer (LC), it has been acknowledged that around 85% of the risk of developing LC is associated with cigarette smoking. Therefore, lung cancer develops in 15% of smokers, suggesting a varying vulnerability to the harmful effects of tobacco carcinogens. Research has indicated that electronic cigarettes (ECs) cause harm to DNA in the lungs and hinder the process of DNA repair in lung tissues. This implicates ECs as a potential cause of lung cancer in mice.

**Table 3:** Comparing findings of studies on the effects of vaping on lung cancer EMT: epithelial-to-mesenchymal transition

Study ID	Study type	Target group	Findings	Interpretation
Tang et al. [36]	Animal	6–8-week-old 85 male mice	Lung adenocarcinoma; bladder urothelial hyperplasia.	e-cigarette smoke exposure induces lung tumour formation in mice
Zahedi et al. [37]	Cell line	A549 CCL-185 cells, which were previously derived from a human lung adenocarcinoma	Enhanced migration of cells	e-cigarettes are capable of causing EMT in a cancer cell line

Every year, about 2.2 million cases of lung cancer are diagnosed in the world, and it ranks second after breast cancer. Electronic cigarettes have risen in popularity in recent years as a means of consuming nicotine or simulating tobacco smoking without carcinogenic combustion products [35]. Most vapers consider smoking electronic cigarettes and/or vaping harmless. Also, some use this type of smoking to reduce the use of tobacco cigarettes. In particular, these smoking systems are common among adolescents and young people due to the availability and lack of regulation of vaping sales. However, are they harmless, and do they affect lung cancer? In 2019, Tang et al. found that exposure to electronic cigarette smoke (ECS) induced lung adenocarcinoma in six- to eight-week-old male mice, suggesting a potential link between ECs and lung cancer [36]. As a result of the study, it was noted that exposure to ECS induces the formation of lung tumours in mice. In the same year, Zahedi et al. demonstrated an epithelial-to-mesenchymal transition (EMT) in lung cancer cells during exposure to e-cigarette products, indicating a possible contribution to cancer progression for those at risk for lung cancer [37].

While studying the effects of vaping on the lungs, we saw a study where smoking e-cigarettes with nicotine was used to reduce the smoking of conventional cigarettes and improve lung health among chronic smokers undergoing a cancer screening programme [38]. Lucchiari et al. showed that 20% of 210 smokers stopped smoking after six months [38]. Surprisingly, there were no side effects at the end of the study, but symptoms such as burning in the throat were found in the subjects after smoking electronic cigarettes. The best preventative measure to curb the adverse health effects associated with smoking is abstaining from smoking or tobacco cessation [39]. Most cancer patients who persist in smoking already recognize the adverse health effects and the importance of stopping smoking [39]. The effects of shaping and e-cigarettes on lung cancer have not been studied as much, although the outcomes can only be guessed. One of the important points is that these types of smoking significantly reduce the functionality of the lungs. As written earlier, exposure to e-cigarettes affects the development of lung adenocarcinoma. Although the carcinogenicity of these tobacco systems has not been fully studied, one can only assume their effect on human health.

Discussion

In recent times, the widespread adoption of vaping, particularly among youth, has been fueled by enticing advertisements, an array of flavours, and aesthetically pleasing designs. Positioned as a tool for quitting traditional smoking, vaping has indeed facilitated smoking cessation for many over the past few decades. However, the ongoing research into the harms of vaping suggests that, while potentially less harmful than traditional cigarettes, vaping is far from being considered a completely safe practice. This discussion sheds light on the adverse effects of vaping on lung health, emphasizing the need for preventive measures.

Pulmonary Impact of Vaping

The impact of vaping on lung health remains an evolving area of investigation, with conclusive results yet to be established. Nonetheless, the documented harm to the lungs associated with vaping is cause for concern. Studies have indicated potential links between vaping and pneumonia, shortness of breath, increased risk of respiratory infections, asthma and bronchitis, and effects on lung tissue [40]. Some studies have shown that vaping or e-cigarettes have the potential to increase susceptibility to pneumococcal infection [41-43]. This inflammation may manifest in symptoms akin to respiratory conditions such as asthma and bronchitis. One of the suggested reasons was the effect of vapour on oxidative stress-induced, PAFR-dependent pneumococcal adhesion to airway epithelial cells and pneumococcal colonization in the mouse nasopharynx [41]. An alarming observation is the onset of lipoid pneumonia in an otherwise healthy patient using cannabis-containing e-cigarettes, suggesting that the composition of vape liquids plays a crucial role in respiratory complications [42]. Some people feel shortness of breath after using a vape [44]. This may be a sign of lung irritation and airway obstruction. Research indicates that vaping may compromise the lung's immune system, heightening the risk of respiratory infections [45]. Some studies have shown that the use of vaping can cause or affect complications such as exacerbations of asthma and bronchitis in some people [46]. Continuous and long-term use of vape may lead to changes in lung tissue. These changes may include inflammation and the destruction of lung tissue.

Risks Associated With Vape Liquid Components

The composition of vape liquids introduces additional considerations for lung health. The inclusion of vitamin E in vape liquids, while commonly used to create vapour in a vape, poses potential risks [47]. The 2019 surge in severe lung diseases reported in individuals using vape liquids containing THC and vitamin E underscores the need for scrutiny [48]. However, the use of vitamin E in vape liquids does not apply to all vape products. These risks are especially related to the use of vitamin E extracted from natural oils that have been illegally added to vape liquids. High-quality, properly refined vitamin E is suggested to pose fewer risks and may even be used as a dietary supplement for lung health. However, research on the long-term effects of PG and glycerin vaporization is inconclusive, raising questions about the safety of these components, especially with excessive or prolonged use [49,50]. This damage can include lung inflammation, increased lung secretions, cough and sputum, lung infection, and respiratory problems such as bronchitis and asthma.

Global Concerns and Regulatory Considerations

In a report, the World Health Organization (WHO) expressed reservations about the widespread use of e-cigarettes, citing concerns about efficacy in smoking cessation and potential harm, especially among youth [PNLP1] [51]. Despite having fewer toxic substances than traditional cigarettes, e-cigarettes are considered harmful. The WHO emphasizes that the high-temperature vaporization of liquid nicotine in e-cigarettes can lead to addiction, warranting vigilant monitoring of these devices.

Long-Term Effects and Comparisons With Smoking

The long-term effects of smoking, including a high risk of stroke, heart disease, and various types of cancer, have been well established [52-54]. According to the report published by the Committee for Disease Control and Prevention (CDC), one out of five people dies due to smoking [55]. Meanwhile, e-cigarettes seem to be a less risky option for people who want to quit smoking. Using an electronic cigarette to quit smoking does not mean that it is safe. Even if the vape liquid does not contain nicotine, its adverse effects may bother the user. To date, little evidence has been provided about the long-term effects of vaping, as it will take at least 10 years to determine the effects of vaping on the lungs. But experience with smoking shows that vaping has similar negative health effects, such as chronic obstructive pulmonary disease (COPD), heart disease, and cancer. Inhaling vape vapour may lead to inflammation in the lungs and cause complications such as cough, phlegm, chest pain, and lung infection [44, 56]. Also, some research has shown that continuous use of vaping may increase the risk of developing lung diseases such as bronchitis and asthma [57]. Some studies have shown that using vapes can increase heart rate, increase blood pressure, and cause heart problems [58]. The use of vape liquids containing nicotine may lead to side effects such as addiction, an increased risk of cardiovascular diseases, nerve disorders, breathing problems, sleep disorders, and an increased risk of cancer such as lung, pancreatic, and brain cancer [58]. The use of vaping by young people and teenagers can lead to the development of nicotine addiction, psychological and cognitive risks, and harmful effects on growth and the brain [59].

Strengths and Limitations

Strengths: The article presents a comprehensive review of existing evidence on the adverse effects of vaping on lung health following PRISMA and Meta-analyses of Observational Studies in Epidemiology (MOOSE) guidelines and the multi-layered approach to searching, which should lend credibility to the methods used. It synthesizes findings from various studies, offering a holistic view of the current state of knowledge on this topic.

The discussion on public health implications goes beyond the scientific findings to highlight the urgent need for public health interventions. This adds a practical dimension to the research by emphasizing the importance of regulatory measures and awareness campaigns.

We made evidence-based recommendations for public health efforts that focused on dispelling misconceptions about the safety of vaping and promoting proven smoking cessation methods. This adds a pragmatic and actionable dimension to the discussion.

We acknowledge the current lack of conclusive evidence on the long-term effects of vaping. This recognition of uncertainty adds credibility to the review and emphasizes the importance of continued research in this evolving field.

By stressing the need for ongoing research to capture emerging trends, the study demonstrates a forward-looking approach. This is crucial in a field where new vaping products and formulations are continuously introduced.

Limitations: Despite the compelling evidence presented, this review acknowledges certain limitations. The review highlights a limitation in the predominantly experimental and case-report nature of the studies reviewed, involving animal or cell line models. While these studies are crucial for understanding biological mechanisms, their findings may not always directly translate to human populations due to species differences in metabolism and physiology. This variability in study designs may pose challenges in establishing causation and generalizing findings to broader populations.

There is a predominance of short-term studies and case reports, which limits the ability to make definitive conclusions about the long-term carcinogenic effects of vaping. Long-term studies are essential to assess the cumulative effects of vaping on lung cancer risk.

Many studies do not adequately control for confounding factors such as concurrent tobacco use, environmental exposures, and genetic predispositions that might influence lung cancer risk, which could lead to conflicting results. Also, significant variability in the types of vaping devices and liquids and the presence of potential contaminants complicate the establishment of a standardized exposure assessment and may lead to conflicting evidence regarding the association between vaping and lung cancer.

Some studies may have small sample sizes, reducing the power to detect significant effects or associations and limiting the generalizability of the findings to a broader population. The studies discuss the use of e-cigarettes as a method to reduce or quit tobacco cigarette smoking, as seen in the study by Lucchiari et al. [38]. However, these findings could be in conflict with the potential harmful effects of vaping, as vaping is sometimes initiated as an alternative to smoking without a full understanding of its risks.

The vaping product market is rapidly evolving, with new products and formulations constantly emerging. This means that the substances and exposures being studied may quickly become outdated, and the findings may not apply to the products currently in use.

Future Directions

Given the points above, future research should prioritize long-term, longitudinal human studies with large and diverse populations, consider the rapidly changing landscape of e-cigarette products, and focus on comprehensive exposure assessments to elucidate the potential risks of vaping, particularly concerning lung cancer. Additionally, clear communication to the public regarding the current understanding and unknowns of vaping risks is important to address misconceptions and inform behaviour.

## Conclusions

Vaping, often regarded as an alternative to smoking, presents its own set of health risks. The review reveals several critical findings: exposure to vaping has been linked to impaired lung function, as evidenced by reduced lung capacity and bronchoconstriction in animal models. Case reports and observational studies indicate a potential association with lung injury, including EVALI, and cell damage indicative of cancerous transformations. While some studies suggest vaping has positioned itself as a smoking cessation aid, this potential benefit is contrasted by the occurrence of throat irritation and other symptoms and does not offset the documented health risks. The World Health Organization (WHO) has expressed concerns regarding the addictiveness of vaping products, particularly among youth, suggesting a need for regulatory oversight. These findings collectively call for robust public health campaigns to correct misconceptions about vaping's safety and emphasize the necessity for continued, comprehensive research into its long-term health impacts.
